# Monitoring Soybean Soil Moisture Content Based on UAV Multispectral and Thermal-Infrared Remote-Sensing Information Fusion

**DOI:** 10.3390/plants13172417

**Published:** 2024-08-29

**Authors:** Hongzhao Shi, Zhiying Liu, Siqi Li, Ming Jin, Zijun Tang, Tao Sun, Xiaochi Liu, Zhijun Li, Fucang Zhang, Youzhen Xiang

**Affiliations:** 1 Key Laboratory of Agricultural Soil and Water Engineering in Arid and Semiarid Areas, Ministry of Education, Northwest A&F University, Xianyang 712100, Chinayouzhenxiang@nwsuaf.edu.cn (Y.X.); 2 Institute of Water-Saving Agriculture in Arid Areas of China, Northwest A&F University, Xianyang 712100, China

**Keywords:** multispectral, thermal infrared, information fusion, soybean, soil moisture content

## Abstract

By integrating the thermal characteristics from thermal-infrared remote sensing with the physiological and structural information of vegetation revealed by multispectral remote sensing, a more comprehensive assessment of the crop soil-moisture-status response can be achieved. In this study, multispectral and thermal-infrared remote-sensing data, along with soil-moisture-content (SMC) samples (0~20 cm, 20~40 cm, and 40~60 cm soil layers), were collected during the flowering stage of soybean. Data sources included vegetation indices, texture features, texture indices, and thermal-infrared vegetation indices. Spectral parameters with a significant correlation level (*p* < 0.01) were selected and input into the model as single- and fuse-input variables. Three machine learning methods, eXtreme Gradient Boosting (XGBoost), Random Forest (RF), and Genetic Algorithm-optimized Backpropagation Neural Network (GA-BP), were utilized to construct prediction models for soybean SMC based on the fusion of UAV multispectral and thermal-infrared remote-sensing information. The results indicated that among the single-input variables, the vegetation indices (VIs) derived from multispectral sensors had the optimal accuracy for monitoring SMC in different soil layers under soybean cultivation. The prediction accuracy was the lowest when using single-texture information, while the combination of texture feature values into new texture indices significantly improved the performance of estimating SMC. The fusion of vegetation indices (VIs), texture indices (TIs), and thermal-infrared vegetation indices (TVIs) provided a better prediction of soybean SMC. The optimal prediction model for SMC in different soil layers under soybean cultivation was constructed based on the input combination of VIs + TIs + TVIs, and XGBoost was identified as the preferred method for soybean SMC monitoring and modeling, with its R^2^ = 0.780, RMSE = 0.437%, and MRE = 1.667% in predicting 0~20 cm SMC. In summary, the fusion of UAV multispectral and thermal-infrared remote-sensing information has good application value in predicting SMC in different soil layers under soybean cultivation. This study can provide technical support for precise management of soybean soil moisture status using the UAV platform.

## 1. Introduction

Soybeans, rich in protein and oil, hold an important position in the trade of grain and oil crops in China [1,2]. They are not only one of the important grain and oil crops, but also an important industrial raw material with a wide range of industrial uses and a huge consumer market [3,4]. Soil is an important natural resource for the survival of living organisms and is closely related to human production, life, and social activities [5,6]. Soil moisture is one of the key components of the Earth’s ecosystem [7], and accurately obtaining the spatiotemporal distribution and variation information of soil moisture is crucial for agricultural production.

Soil moisture in the field is closely related to crop growth, and plays a key role in the growth and development of crops [8,9]. Accurate estimation of soil moisture content (SMC) in the field is of great significance for predicting crop yield [10], field water stress [11], and crop growth conditions [12]. Insufficient SMC may lead to water stress in crops, resulting in conditions such as wilting, slow growth, or even death [13], while excessive soil moisture can lead to root diseases and hypoxia [14], affecting the normal development of crops. Therefore, monitoring SMC throughout the entire crop growth cycle is crucial, as it can reveal details of the crop’s water and nutrient status, potential environmental stresses, and the interaction between crops and the soil environment [15], which may vary in different geographical locations and under different climatic conditions. Soil moisture monitoring can help farmers or growers to detect potential water-stress issues in a timely manner and take timely irrigation or drainage measures [16]. Visually, the crop’s response to water stress may not be apparent in the early stages, especially in large areas of farmland where subtle changes are difficult to detect with the naked eye. Furthermore, conventional techniques for assessing soil moisture levels, including the gravimetric technique and the Time Domain Reflectometry (TDR) method [17], although accurate, are often time-consuming and require destructive sampling, which limits their scalability and real-time applicability in large areas of farmland [18]. Therefore, traditional methods may not be suitable for real-time monitoring and rapid response of soil moisture conditions in precision agriculture.

UAV systems equipped with various portable sensors have become widely used tools in crop monitoring, due to their ability to quickly and efficiently obtain high-resolution remote-sensing images that reflect the growth status of crops [19]. This technology can more quickly and comprehensively obtain information about crop health, growth trends, and environmental variables, thereby helping farmers and researchers make wiser decisions to improve crop productivity, resource efficiency, and overall agricultural sustainability [20]. In recent years, the application of UAV multispectral sensors in precision agriculture has been continuously expanding, including estimating crop leaf-nitrogen content [21], aboveground biomass [22], chlorophyll content [23], and SMC [24]. Vegetation indices extracted from UAV multispectral images have great potential for monitoring SMC, which enhances the ability to reflect crop growth conditions through mathematical methods such as summation, ratio, and normalization of the spectral reflectance of the crop canopy [25], reducing the impact of soil, noise, and other external environmental factors [26], providing an effective method for monitoring SMC. In addition, texture features, because of their ability to reflect the crop canopy and distribution patterns [27], are often combined with vegetation indices to improve the accuracy of predicting crop parameters. Yang et al. (2024) used vegetation indices, texture features, and texture indices to predict the water content of soybean leaves, and found that using the complementary information of vegetation indices and texture can significantly improve the prediction accuracy of the model [28]. Thermal-infrared sensors, because of their ability to directly reflect the temperature of the crop canopy, are closely related to crop water stress and the degree of soil drought [29]. The strategy of fusing thermal-infrared and multispectral data has been proven to significantly enhance the accuracy of predicting water-stress indicators such as SMC [30], leaf water content [31], and canopy transpiration and soil evaporation [32]. By integrating the thermal characteristics of thermal-infrared remote sensing with the physiological and structural information of vegetation revealed by multispectral remote sensing, a more comprehensive understanding of the crop’s response to moisture conditions can be achieved [33]. Peng et al. (2022) used vegetation indices, coverage, and canopy temperature to establish a model for monitoring the moisture status of grape plants, and the model established with the combination of multispectral and thermal-infrared information as predictive variables was superior to the variable combination using single-sensor information [34].

During the flowering stage of soybeans, we collected multispectral and thermal-infrared remote-sensing information, as well as soil moisture-content sample data. During the flowering stage of soybean, the canopy coverage is high, allowing the inference of plant water-stress conditions through analysis of spectral data from the canopy. The spectral response of plants under water stress can be quantified using vegetation indices and texture features. Furthermore, although optical data cannot directly penetrate deep soil layers, the distribution of plant roots in the shallow soil enables the plant’s water status to indirectly reflect the moisture conditions of deeper soil layers through its canopy spectral characteristics. This indirect relationship is well-documented in agricultural and vegetation research. This study aims to explore the synergistic effects and monitoring capabilities of multispectral remote-sensing information and thermal-infrared information in predicting soil moisture content in soybeans. The specific purpose of the study is (1) determining the optimal combination of multispectral and thermal-infrared information; and (2) combining machine learning algorithms to construct the optimal prediction model for soybean soil-moisture content using remote-sensing information-fusion technology. By optimizing the combination of multispectral and thermal-infrared information, this study not only improves the accuracy of soil-moisture-content prediction but also provides an efficient monitoring tool for agricultural soil moisture management. This is of significant practical value for guiding precision irrigation, optimizing water resource utilization, and increasing crop yields.

## 2. Materials and Methods

### 2.1. Research Area and Test Design

**Figure 1:**
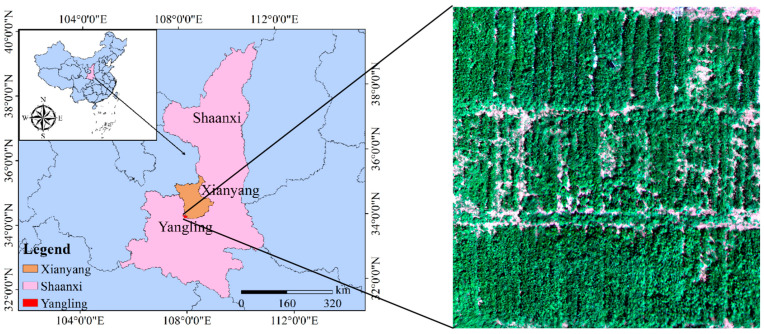
Research area overview and aerial photography of some fields in the research area.

The experiment was conducted in the loess soil of the experimental field at the Water-saving Irrigation Test Station of the Key Laboratory of Agricultural Soil and Water Engineering in Arid and Semiarid Areas of the Ministry of Education, Northwest A&F University, Yangling, Shaanxi (Figure 1). Situated at the geographic coordinates of 108°24′ east longitude and 34°20′ north latitude, the experimental site is perched at an elevation of 524.7 m above sea level. It falls within the warm temperate monsoon climate category, characterized by a semi-humid region where the bulk of the precipitation occurs during the months of July to September. The long-term precipitation average stands at 580 mm, while the average rate of evaporation is notably higher, reaching 1500 mm. The region experiences a mean annual temperature of 12.9 degrees Celsius. The soil’s field capacity to retain water within the top 100 cm of the soil profile is between 23% and 25%, with the moisture level at which plants begin to wilt being 8.5% by mass. In the uppermost 20 cm of the soil, the pH level is measured at 8.14, enriched with organic matter at a concentration of 12.0 g/kg. The soil also contains total phosphorus at 0.60 g/kg, with 8.21 mg/kg available for plant uptake. Potassium levels are substantial, with a total content of 14.10 g/kg and a rapidly available fraction of 131.97 mg/kg. The total nitrogen content is recorded at 0.89 g/kg, complemented by an alkaline hydrolyzable nitrogen fraction of 55.30 mg/kg.

### 2.2. Experimental Design

This experiment was designed with four nitrogen application rates: N0 (0 kg N/ha), N1 (60 kg N/ha), N2 (120 kg N/ha), and N3 (180 kg N/ha). Additionally, four types of mulching were implemented: Straw mulching (SM), Straw and film mulching (SFM), Film Mulching (FM), and No mulching (NM), resulting in a total of 16 treatments with three replicates each. The plot area for each was 4 m by 6 m, equating to 24 m^2^, and plots were arranged randomly with a 2 m protective zone around the experimental area. Phosphorus and potassium supplements were uniformly distributed across the experimental plots at a dosage of 30 kg/ha. The nitrogen input was facilitated through urea, which contains 46% nitrogen. The phosphorus source was in the form of superphosphate, comprising 16% phosphorus, while the potassium was supplied by potassium chloride, with a concentration of 62% potassium. The application of these fertilizers took place in trenches positioned 25 cm from the crop line, coinciding with the pre-sowing phase. Concurrently, the straw mulching amount was 9000 kg/ha. The cultivation of soybeans was executed with a density of 300,000 plants per hectare, arranged with a row spacing of 50 cm and an inter-plant distance of 10 cm. The soybean variety is Shanning 17. Soybeans were sown on 14 June 2023, and 4 June 2024, and harvested on 29 September 2023; the 2024 crop had not been harvested at the time of writing. Other field management practices (such as pesticide application and weeding) were consistent with local standards. Remote-sensing and ground data were collected on 4 August 2023, and 31 July 2024, corresponding to the flowering stage of the soybean.

### 2.3. Measurement Items and Methods

#### 2.3.1. Remote-Sensing Data Acquisition

Data acquisition via unmanned aerial vehicle (UAV) remote sensing was facilitated by the Matrice600Pro hexacopter (DJI, Shenzhen, China), a model engineered by DJI. This UAV boasts a substantial maximum payload capacity of 6 kg and can sustain flight operations for a duration ranging from 25 to 35 min. The Matrice600Pro was outfitted with a duo of advanced sensors designed for capturing imagery: a thermal-infrared imaging sensor branded as ZENMUSE XT (DJI, Shenzhen, China) and a multispectral sensor known as Yusense MS600 (Yusense Information Technology and Equipment (Qingdao) Co., Ltd., Qingdao, China). The aerial missions were strategically scheduled on days characterized by the absence of wind and the presence of clear, sunny skies, to ensure optimal conditions for data collection. The UAV flew at a height of 20 m for all flight missions, and the MS600 multispectral camera was configured with six spectral bands for spectral information collection, namely 490 nm, 555 nm, 680 nm, 720 nm, 800 nm, and 900 nm, with a resolution of 1 cm. The thermal-infrared camera had a spectral range of 7.5~13.5 μm and a resolution of 0.05 °C, and images were taken with the camera lens perpendicular to the ground during the flight. To ensure that our estimation of soil moisture content (SMC) is minimally affected by field cover methods, we implemented the following measures: first, we conducted extensive data collection under different cover conditions, including spectral data collection under various cover materials and vegetation coverage levels within the same growth stage. Second, during the data analysis phase, we paid special attention to the impact of cover conditions on spectral and thermal-infrared data, employing advanced data processing techniques, such as spectral feature extraction in ENVI software, to distinguish and extract information related solely to the plant canopy. This allowed us to eliminate the influence of cover factors from spectral data.

#### 2.3.2. Ground Data Collection

Ground data were collected simultaneously with the remote-sensing data, mainly including the collection of soil moisture content (SMC) and canopy temperature in each plot. At the flowering stage of soybean, the range of root zone is 0~60 cm. Soil moisture was measured using the oven-drying method, determining the water content *W* of the soil in the 0~20 cm, 20~40 cm, and 40~60 cm soil layers under soybean cultivation within each treatment plot. Five points were selected in each plot, using the five-point sampling method, and soil samples were collected at each point with an auger and collected in aluminum boxes, with the average of five sets of data taken as the final value. The wet weight *M1* of the soil samples (less than 100 g) was promptly weighed using a balance with a sensitivity of 0.01 g, and then the soil samples were dried in an oven at 105 °C for 6~8 h until a constant weight was reached, with the oven temperature maintained between 100 and 110 °C. The dry weight *M2* of the soil samples was then measured, and the SMC was calculated using the following formula:

(1) $$W=\frac{M1-M2}{M1}$$

**Figure 2:**
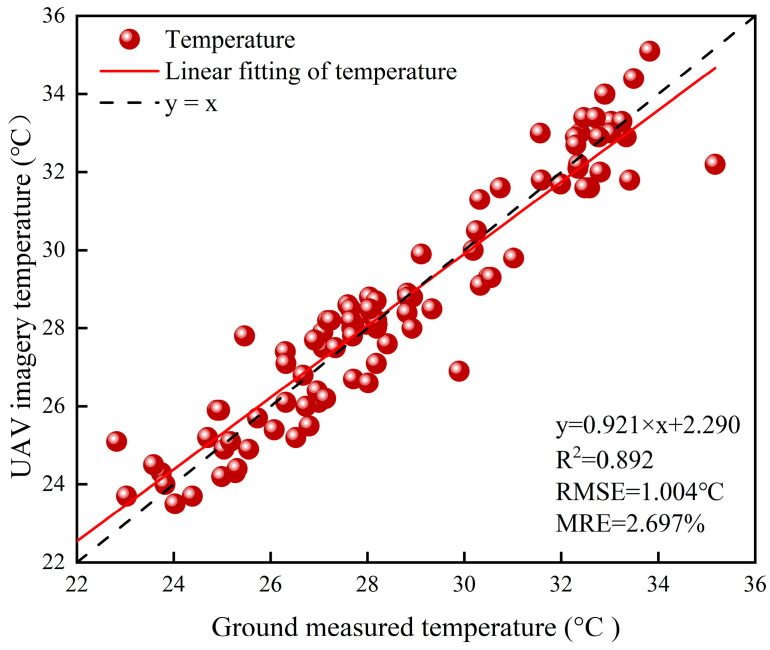
Scatter plot of canopy temperature extracted from thermal-infrared images and actual canopy temperature.

Canopy temperature was measured using a handheld thermal-infrared thermometer with a thermal accuracy of ±1 °C. In each experimental plot, six plants were selected, and the average canopy temperature of the six plants was taken as the canopy temperature of the soybean in that plot. While collecting canopy temperature, the temperature of the water in a bucket placed in advance in the experimental area was also collected. The thermal-infrared images collected by the UAV were input into the FLIR Tools V3.1.1 software, and the average leaf temperature and water temperature were used as reference temperatures to calibrate the pixel temperatures in the thermal-infrared images. The canopy temperature at different times in each plot was extracted, and the measured value of the handheld thermometer was used as the reference value for the canopy temperature of the soybean, to carry out the analysis of the results and accuracy of the thermal-infrared image temperature and the actual measured canopy temperature. Figure 2 shows the correlation between the canopy temperature extracted from the thermal-infrared image and the actual canopy temperature at the flowering stage of the soybean, with an R^2^ of 0.892, and RMSE and MRE of 1.004 °C and 2.697%, respectively. The high accuracy and good fit indicate a strong correlation between the thermal-infrared image temperature and the actual measured canopy temperature.

#### 2.3.3. Preprocessing of Multi-Source Remote-Sensing Data

In this study, the Yusense Map 2.2.2 software was used to stitch the UAV multispectral images, with automatic geometric and radiometric correction preprocessing during the stitching process. The ENVI 5.3 software was used to perform mask processing on the shadows and soil background in each plot of the stitched images, based on the threshold method, extracting the spectral reflectance of each plot and each band. Vegetation indices were obtained through the combination of the extracted single-band spectral reflectance. The specific vegetation indices selected are shown in Table 1.

The Gray-Level Co-occurrence Matrix (GLCM) stands as a prevalent technique for texture analysis, currently among the most frequently utilized in the field. Characterized by its robustness to image rotation, capability to capture multi-scale characteristics, and relatively low computational demand, the GLCM has found extensive applications across various domains, including image processing, pattern recognition, and remote-sensing observation. The texture attributes derived from this method encompass a spectrum of statistical measures such as Mean, Variance, Homogeneity, Contrast, Dissimilarity, Entropy, Second Moment, and Correlation, as detailed in Table 2. In ENVI 5.3 (Harris, Bloomfield, CO, USA), GLCM is used to perform a 3 × 3 sliding filter on the grayscale images of RGB images to extract 8 texture features in the directions of 0°, 45°, 90°, and 135°. The average values of the 4 directions are taken as the final texture features. In the ENVI 5.3 software, texture-feature images of each band are delineated for sampling areas of each plot, the average texture values of the delineated areas are extracted, and these values are used as the texture-feature values for the plot.

This study also utilized texture features to construct texture indices. Six types of texture indices (*TI*) were defined as follows: Normalized difference texture index (*NDTI*), Difference texture index (*DTI*), Ratio texture index (*RTI*), Nonlinear texture index (*NTI*), Reciprocal difference texture index (*RDTI*), and Reciprocal additive texture index (*RATI*). All possible combinations of measurements were made by combining six bands (490, 555, 680, 720, 800, and 900 nm) with eight GLCM-based texture-feature values, to explore their estimation capabilities.

(2) $$NDTI=\frac{T1-T2}{T1+T2}$$

(3) DTI=T1−T2

(4) RTI=T1/T2

(5) $$NTI=\frac{T1^{2}-T2}{T1^{2}+T2}$$

(6) RDTI=1/T1−1/T2

(7) RATI=1/T1+1/T2

In the formulas mentioned, *T*1 and *T*2 represent any two different texture features based on the Gray-Level Co-occurrence Matrix (GLCM).

Thermal-infrared technology is widely applied in the study of crop water stress, the monitoring of infectious diseases, frost damage, and yield prediction. In this study, canopy temperature (*T_C_*) was extracted from thermal-infrared images, and four thermal-infrared indices were calculated based on this, namely Canopy–Air Temperature Difference (*T_C_D*), Normalized Canopy Temperature (*NRCT*), Canopy Relative-Temperature Difference (*CRTD*), and Soil Relative-Temperature Difference (*SRTD*). The specific formulas are as follows:

(8) $$T_{c}D=T_{\mathrm{ci}}-T_{c}$$

(9) $$NRCT=(T_{\mathrm{ci}}-T_{\mathrm{cmin}})/(T_{\mathrm{cmax}}-T_{\mathrm{cmin}})$$

(10) $$CRTD=(T_{c\max}-T_{\mathrm{cmin}})/(T_{\mathrm{cmax}}+T_{\mathrm{cmin}})$$

(11) $$SRTD=(T_{s\max}-T_{\mathrm{smin}})/(T_{\mathrm{smax}}+T_{\mathrm{smin}})$$

In this formula, $T_{c}$ represents the air temperature, $T_{ci}$ is the canopy temperature of the i-th pixel in the image, $T_{c\max}$ is the maximum temperature measured across the entire experimental field, $T_{c\min}$ is the minimum temperature measured across the entire experimental field, $T_{s\max}$ is the maximum soil temperature within each sampled plot, and $T_{s\min}$ is the minimum soil temperature within each sampled plot.

#### 2.3.4. Model Construction and Validation

This study collected multispectral and thermal-infrared remote-sensing data, as well as SMC sample data during the flowering stage of soybean, comprising a total of 96 data sets. Two-thirds of the data samples were used as the model training set, and one-third as the model validation set. The data sources included Vegetation Indices (VIs), Texture Features (TF), Texture Indices (TIs), and Thermal-Infrared Vegetation Indices (TVIs). Spectral parameters with a correlation significant at the *p* < 0.01 level were selected and input into the model as single- and fused-input variables. In the quest to develop predictive models for soybean soil-moisture content (SMC), we leveraged a trio of sophisticated machine learning techniques, namely eXtreme Gradient Boosting (XGBoost), Random Forest (RF), and a Backpropagation Neural Network (BP) enhanced by a Genetic Algorithm (GA-BP). The XGBoost technique underwent a meticulous parameter tuning process using a grid search approach, culminating in the selection of 100 boosting stages (n_estimators), a learning rate set to 0.03, and a cap on the tree depth at 5, as per the optimized parameters [48]. The implementation of the RF model required the construction of a large number of decision trees and the use of variable swapping and alteration to enhance predictive performance; after multiple training sessions and error analyses, the number of decision trees was set to 100 [49]. The genetic algorithm optimized the initial weights of the BP neural network, replacing the gradient descent process for adjusting network weights and thresholds. The genetic algorithm started with a population size of 5, set for 50 generations, with a crossover probability of 0.4% and a mutation probability of 0.05%; after optimization, the BP neural network had 5 nodes in the input layer, 5 nodes in the hidden layer, 1 node in the output layer, a maximum of 1000 iterations, and a training target of 1 × 10^−6^ [50]. All algorithmic modeling in this study was completed in MATLAB R2022a software.

The accuracy and reliability of the model’s fit were assessed through key performance indicators such as the Coefficient of Determination (R^2^), the Root Mean Square Error (RMSE), and the Mean Relative Error (MRE). A high R^2^ value, nearing the threshold of 1, denotes a model with exceptional predictive capabilities. Furthermore, minimal RMSE and MRE values suggest a model that exhibits consistent performance and delivers predictions that are closely aligned with one another. The calculation formulas are as follows:

(12) $$R^{2}=\frac{\sum_{i=1}^{n}{\left({\hat{y}}_{l}-\bar{y}\right)}^{2}}{\sum_{i=1}^{n}{\left(y_{i}-\bar{y}\right)}^{2}}$$

(13) $$RMSE=\sqrt{\frac{\sum_{i=1}^{n}{\left(y_{i}-\bar{y}\right)}^{2}}{n}}$$

(14) $$MRE=\frac{1}{n}\sum_{i=1}^{n}\frac{\left|{\hat{y}}_{i}-y_{i}\right|}{y_{i}}\times 100\%$$

In the formula $\hat{y_{i}}$—Model predictive values:

$y_{i}$—Actual sampling value;

$\bar{y}$—Actual sampling value;

*n*—number of samples.

#### 2.3.5. Methodology

**Figure 3:**
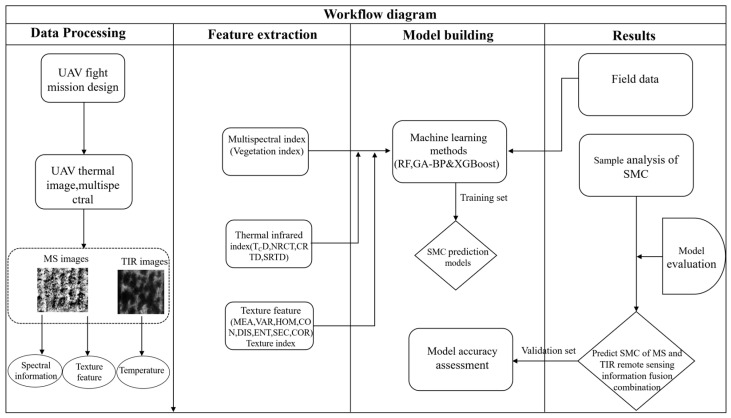
Flowchart of the proposed method.

To explore the synergistic effect and monitoring capability of multispectral remote-sensing information and thermal-infrared information in predicting SMC, this study collected multispectral and thermal-infrared remote-sensing information and SMC sample data during the flowering stage of soybean. The data sources included vegetation indices (VIs), texture features (TF), texture indices (TIs), and thermal-infrared vegetation indices (TVIs). Spectral parameters with a significant correlation level (*p* < 0.01) were selected and input into the model as single- and fused-input variables. In this research, a trio of advanced machine learning algorithms was employed to develop predictive models for soybean soil-moisture content (SMC). These included eXtreme Gradient Boosting (XGBoost), the ensemble method known as Random Forest (RF), and a Backpropagation Neural Network (BP) enhanced by a Genetic Algorithm (GA). The models were formulated utilizing the integrated data acquired from unmanned aerial vehicle (UAV)-mounted multispectral and thermal-infrared sensors. A visual representation of the workflow and analytical process is depicted in Figure 3, illustrating the sequence of data handling and the methodologies applied throughout the study.

## 3. Results

### 3.1. Selection of Spectral Indices and Textural Features

To fully exploit the spectral information contained within multispectral data, this study selected 15 commonly used vegetation indices (Table 1) and analyzed their correlation with the soil moisture content (SMC) at different soil layers under soybean cultivation. The vegetation indices with correlations significant at the *p* < 0.01 level are presented in Table 3. Significant correlations (*p* < 0.01) with SMC were found for indices across various soil layers under soybean cultivation. Notably, the MSR, DVI, GCVI, and NLI all demonstrated strong correlations, with MSR showing the highest correlation coefficients of −0.661, −0.657, and −0.510 for the 0~20 cm, 20~40 cm, and 40~60 cm soil layers, respectively. Additionally, the study calculated the correlation between texture features and the SMC at different soil layers under soybean cultivation (Table 4). The texture features with the highest correlation coefficients in the 0~20 cm, 20~40 cm, and 40~60 cm soil layers under soybean cultivation were the Second Moment of band 5, and the mean values of bands 2 and 3, with correlation coefficients of 0.585, 0.644, and 0.519, respectively.

This comprehensive analysis ensures the selection of the most relevant spectral and textural indicators for accurate estimation of SMC, which is crucial for the development of robust predictive models in agricultural and environmental studies.

### 3.2. Texture-Feature-Index Construction

**Figure 4:**
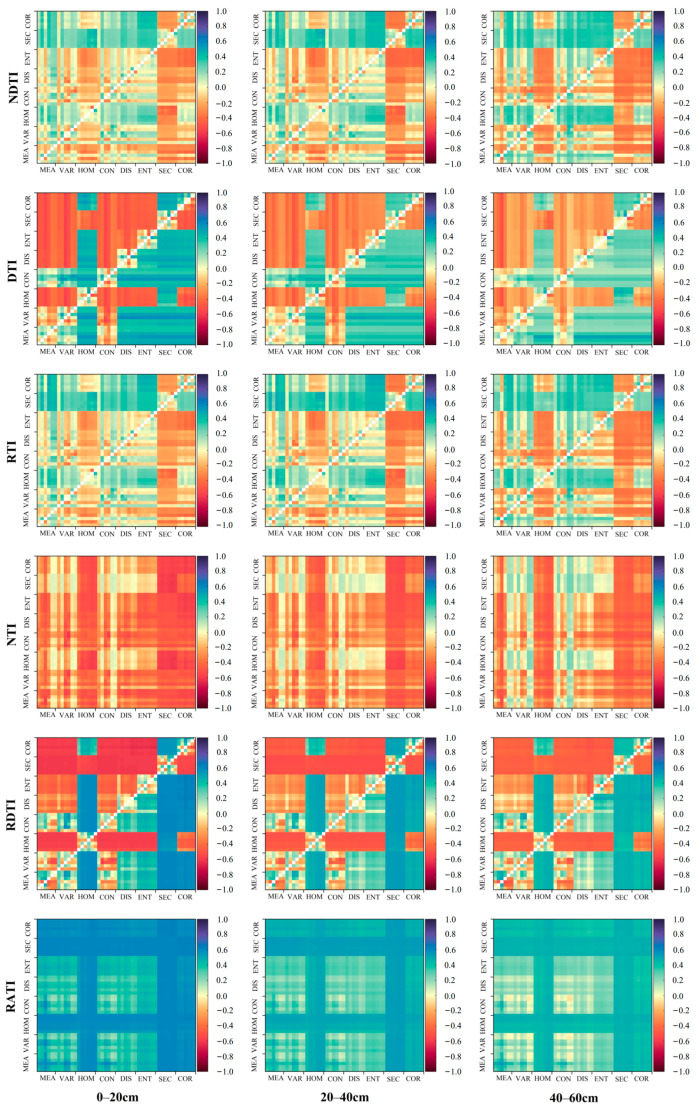
Correlation matrix between soybean SMC at different soil layers under soybean cultivation and randomly combined texture indices.

To enhance the relevance of texture features, we extracted six different texture indices through random combinations of various texture features (Table 5 and Figure 4). After screening, the correlations between the randomly combined texture indices and the SMC at different soil layers under soybean cultivation all reached a highly significant level (*p* < 0.01). Overall, the Reciprocal Difference Texture Index (RDTI) was the most relevant texture index for the SMC in the 0~20 cm soil layer of soybean, with a correlation coefficient of 0.646, and the texture combination was (Cor3, Ent5). The Nonlinear Texture Index (NTI) was the most relevant texture index for the SMC in the 20~40 cm and 40~60 cm soil layers under soybean cultivation, with correlation coefficients of −0.583 and −0.550, respectively, and the texture combinations were (Sec5, Hom5) and (Mea3, Mea2).

### 3.3. Selection of Thermal-Infrared Vegetation Indices

**Figure 5:**
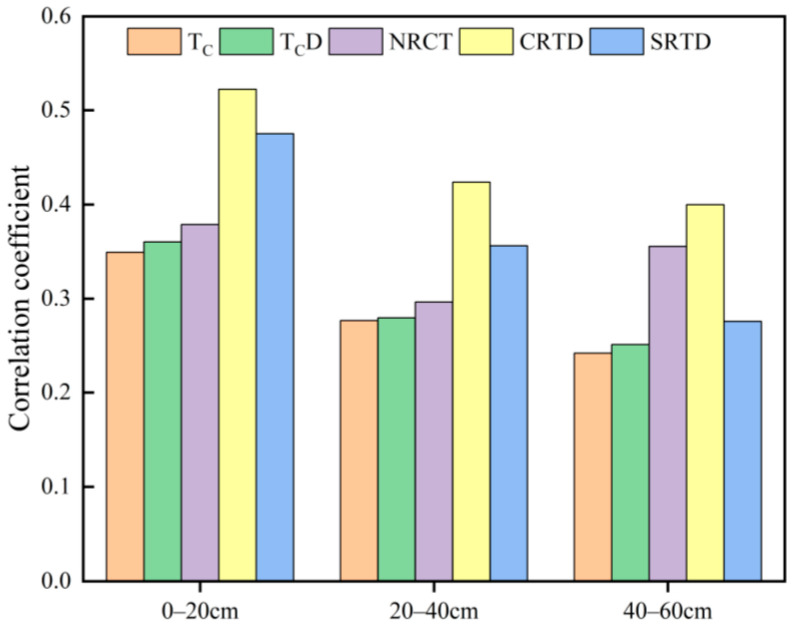
Correlation between thermal-infrared indices and SMC in different soil layers under soybean cultivation.

In this study, canopy temperature (T_C_) was extracted from thermal-infrared images, and four thermal-infrared vegetation indices were calculated based on this: Canopy–Air Temperature Difference (T_C_D), Normalized Canopy Temperature (NRCT), Canopy Relative-Temperature Difference (CRTD), and Soil Relative-Temperature Difference (SRTD). The correlation between these five indices and the SMC at different soil layers under soybean cultivation was analyzed (Figure 5). The results indicated that CRTD and SRTD had a better correlation with the SMC of soybean, with both correlations being at a highly significant level (*p* < 0.01). The correlation of the thermal-infrared indices constructed at different soil layers under soybean cultivation with SMC showed a trend of 0~20 cm > 20~40 cm > 40~60 cm.

### 3.4. Soybean Soil-Moisture Content (SMC) Prediction Models Based on Multispectral, Thermal-Infrared, and Multispectral Thermal-Infrared Remote-Sensing Information Fusion

**Figure 6:**
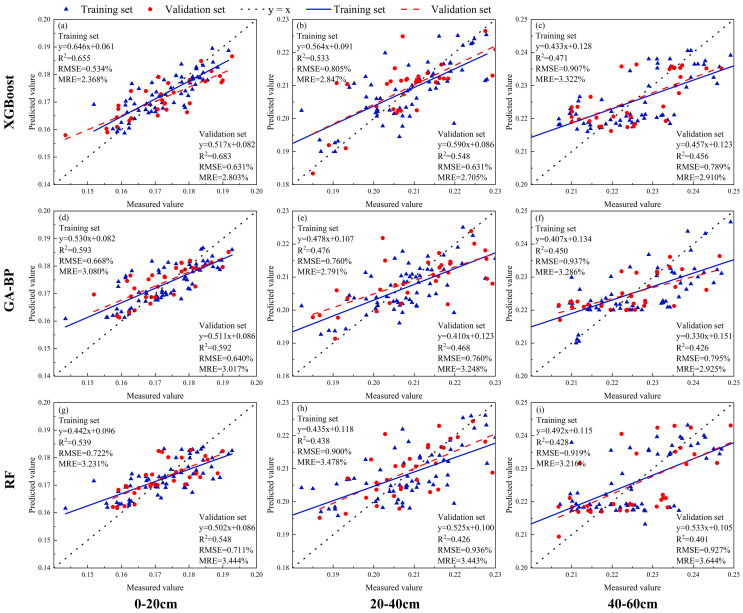
Optimal prediction models for SMC in different soil layers under soybean cultivation based on vegetation indices (VIs) with XGBoost, GA-BP, and RF ((**a**–**i**) represents the soil moisture content of 0–20 cm, 20–40 cm, 40–60 cm soil layers under soybean cultivation using XGBoost, GA-BP and RF models based on vegetation index (VIs) input variables).

This study collected multispectral and thermal-infrared remote-sensing information during the flowering stage of soybean, and extracted four types of model input sources: vegetation indices, texture features, texture indices, and thermal-infrared vegetation indices. The models were assessed with both single-input variables and fused-input variables. When evaluating the performance of the models with single-input variables (Table 6), Vegetation Indices (VIs) were found to be the optimal single-model input, with all four models achieving the highest accuracy and good fit (Figure 6). The XGBoost model performed the best, with an R^2^ range of 0.456 to 0.683 in the validation set, an RMSE range of 0.631 to 0.795%, and an MRE range of 2.910 to 3.444%.

**Figure 7:**
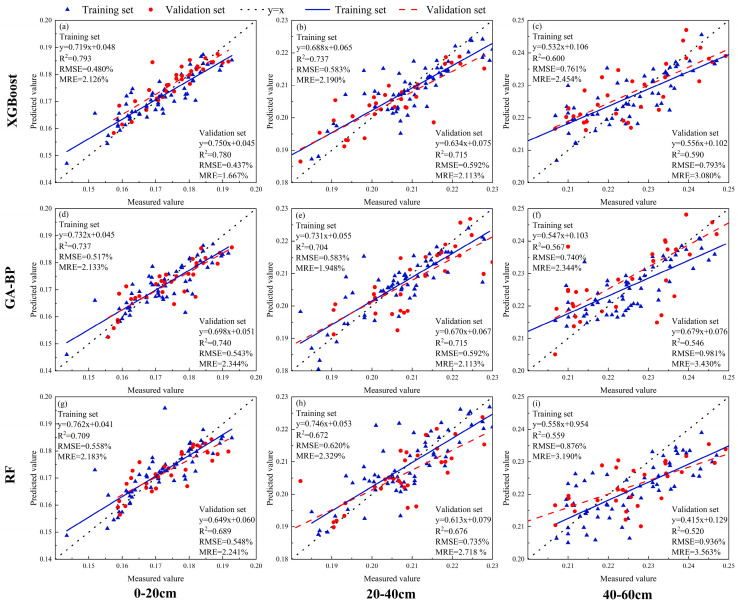
Optimal prediction models for SMC in different soil layers under soybean cultivation based on the combination of vegetation indices (VIs), texture indices (TIs), and thermal-infrared vegetation indices (TVIs) using XGBoost, GA-BP, and RF ((**a**–**i**) represents the soil moisture content of 0–20 cm, 20–40 cm, 40–60 cm soil layers under soybean cultivation using XGBoost, GA-BP and RF models based on the combination of vegetation indices (VIs), texture indices (TIs), and thermal-infrared vegetation indices (TVIs) input variables).

The fused-input variables were input into the model, and their model performance was assessed (Table 7). The combination of Vegetation Indices (VIs), Texture Features (TIs), and Thermal-Infrared Vegetation Indices (TVIs) proved to be the optimal fused-model input, with all four models achieving the highest accuracy and good fit (Figure 7). The XGBoost model also performed the best among them, with an R^2^ range of 0.589 to 0.780 in the validation set, an RMSE range of 0.437 to 0.793%, and an MRE range of 1.667 to 3.080%. The performance of different models can be represented as XGBoost > GA-BP > RF. Moreover, compared to the 40~60 cm soil layer, these models demonstrated better predictive performance in the 0~20 cm and 20~40 cm soil layers.

## 4. Discussion

Multispectral and thermal-infrared sensors, due to their complementary information, often integrate the information from both types of sensors for estimating crop-growth physiological indicators and assessing water and nutrient status in agricultural remote sensing [51]. This study acquired remote-sensing information from different sensors to assess and predict the soil moisture-content (SMC) status of soybean. The results showed that the vegetation indices (VIs) based on multispectral sensors had the best accuracy in monitoring SMC in different soil layers under soybean cultivation, with a determination coefficient ranging from 0.456 to 0.683. Vegetation indices can fully utilize the rich information of multispectral data, reduce the impact of soil information on SMC, eliminate redundant information, and reduce the complexity of the model [52]. Previous studies have shown that vegetation indices such as the Normalized Difference Vegetation Index (NDVI), Modified Simple Ratio (MSR), and Triangular Vegetation Index (TVI), built based on multispectral information, can greatly preserve the spectral characteristics of the vegetation canopy and can assess well the water stress of crops like winter wheat [53] and rapeseed [17]. The thermal-infrared vegetation indices (TVIs) based on thermal-infrared sensors also show good results in soil moisture monitoring, with a determination coefficient ranging from 0.384 to 0.601. The information from thermal-infrared sensors can directly reflect the crop canopy temperature, which is closely related to the water status of the crop and can characterize well the water stress of the crop [54]. Marques et al. (2023) harnessed aerial imagery obtained from Unmanned Aerial Vehicles (UAVs) to forecast critical indicators of plant water stress, which included relative water content (RWC), midday leaf-water potential (Ψ_MD_), and stomatal conductance (gs). Their findings underscored the substantial efficacy of thermal vegetation indices in the evaluation of these stress indicators [31]. When using single-texture information, the prediction accuracy is the worst, but by combining texture-feature values to obtain new texture indices, the performance of estimating SMC by texture features can be effectively improved. This is because combining texture-feature values through normalization, difference, ratio, and other methods can reduce the impact of soil background, solar angle, and sensor view angle, amplify the subtle differences between object spectral features, and highlight object features [55].

When using two input variables to predict the SMC status of different soil layers under soybean cultivation, the vegetation index combined with texture index (VIs + TIs) based on multispectral sensors achieved the best accuracy. After the vegetation index is combined with texture information, it can not only represent the canopy structure of the crop, but also reduce the impact of “same spectrum different objects” and “same object different spectrum”, thereby improving the prediction accuracy of crop growth parameters, to a certain extent [56]. When predicting the soil moisture status of different soil layers under soybean cultivation based on the input variables of Vis + TVIs, the accuracy is lower, but when the texture index (TIs) is added, it makes up for this deficiency and greatly enhances the prediction accuracy, with an R^2^ range of 0.589 to 0.780. This may be because the texture information extracted from the multispectral sensor includes information about the growth and structure of the crop canopy, which can overcome the inherent saturation problem of spectral information, to a certain extent [57]. Among the three models in this study, the fusion of vegetation index, texture index, and thermal features is better than any two combinations of information. These results indicate that spectral information, texture features, and thermal features provide special supplementary information that helps to predict crop soil moisture. The results indicate that water-stress conditions in soybean can be effectively monitored through vegetation indices and texture features. Additionally, surface temperature captured by thermal-infrared data serves as an indicator of plant water stress, providing supplementary information for assessing plant water status.

Among the three models selected in this study, the XGBoost-based model has the highest accuracy, indicating that XGBoost has a significant advantage in predicting crop SMC, which is consistent with previous studies [58]. XGBoost (Extreme Gradient Boosting) is an efficient gradient-boosting framework that uses multi-threading and distributed computing, can handle large-scale datasets under limited memory conditions, and can remove content that contributes less to the model during the operation, enhancing the interpretability of the model. Although GA-BP shows excellent performance in tasks such as image and speech recognition, it is not efficient and accurate enough in regression and classification tasks. RF, although providing good model robustness and the ability to handle feature interaction, may encounter performance bottlenecks when dealing with large-scale datasets. Based on the input combination of VIs + TIs + TVIs, this study constructed the optimal prediction model for SMC in different soil layers under soybean cultivation, and XGBoost can be considered as the preferred method for soybean SMC monitoring and modeling. In addition, compared with the 40~60 cm soil layer, these models show better predictive performance in the 0~20 cm and 20~40 cm soil layers. This study can provide real-time and efficient technical services for monitoring the surplus and deficit status of crop soil moisture in practical applications.

Building upon the success of our UAV-based multispectral and thermal-infrared remote-sensing research, the future of our work will innovate in collecting a diverse dataset to enhance model adaptability, explore cutting-edge algorithms to refine predictive accuracy, and develop an ensemble of models for robust forecasting. We will implement a real-time monitoring system to facilitate proactive agricultural management, collaborate with a broad spectrum of disciplines to understand soil moisture’s impact on crop health, and work towards commercializing our technology to make precision farming more accessible. We will assess the economic and environmental sustainability of our approach, ensuring it is scalable and adaptable to various farming practices. Additionally, we aim to promote sustainable agricultural practices, all while ensuring our research remains at the forefront of innovation in precision agriculture and sustainable development.

## 5. Conclusions

This investigation amassed a rich dataset during the flowering stage of soybean cultivation, integrating both multispectral and thermal-infrared remote-sensing measurements along with soil moisture-content (SMC) samples. The dataset was enriched with a spectrum of indicators, including vegetation indices (VIs), texture feature indices (TFs) derived from textural analysis, and thermal-infrared vegetation indices (TVIs). Spectral parameters that demonstrated significant correlations at the stringent *p*-value threshold of less than 0.01 were identified and utilized as single and fused inputs for model formulation. The construction of predictive models for soybean SMC was accomplished by engaging three sophisticated machine learning methodologies: eXtreme Gradient Boosting (XGBoost), Random Forest (RF), and a Backpropagation Neural Network (BP) optimized by a Genetic Algorithm (GA-BP). These models were adeptly tailored to harness the synergistic potential of UAV-acquired multispectral and thermal-infrared remote-sensing data. The conclusions are as follows:

(1) Among the single-input variables, the vegetation indices (VIs) derived from multispectral sensors demonstrated the highest accuracy in monitoring SMC across different soil layers under soybean cultivation. The predictive accuracy was the poorest when using single-texture information alone, but by combining texture-feature values to create new texture indices, the performance of estimating SMC was effectively enhanced. The fusion of vegetation indices (VIs), texture indices (TIs), and thermal-infrared vegetation indices (TVIs) provided a better prediction of soybean SMC.
(2) Based on the input combination of VIs, TIs, and TVIs, this study constructed the optimal prediction model for SMC in different soil layers under soybean cultivation, with XGBoost being the preferred method for soybean SMC monitoring and modeling. Moreover, compared to the 40~60 cm soil layer, these models exhibited superior predictive performance in the 0~20 cm and 20~40 cm soil layers.

In summary, the results of this study demonstrate the effectiveness of multi-spectral and thermal-infrared data in monitoring SMC under specific agricultural management practices. This research provides a new perspective and method for monitoring water stress in soybean, which has significant practical application value for agricultural water management.

## Figures and Tables

**Table 1:** Selected vegetation index.

Vegetation Index	Computational Formula	References
Modified Triangular Vegetation Index (MTVI)	$1.2(1.2(R_{NIR}-R_{G})-2.5(R_{RE}-R_{G}))$	[35]
Soil-Adjusted Vegetation Index (SAVI)	$1.5(R_{NIR}-R_{R})/(R_{NIR}+R_{R}+0.5)$	[36]
Optimized Soil-Adjusted Vegetation Index (OSAVI)	$(1+X)\frac{R_{NIR}-R_{G}}{R_{NIR}+R_{G}+X}$	[37]
Modified Soil-Adjusted Vegetation Index (MSAVI)	$[(2R_{NIR}+1-\sqrt{{\left(2R_{NIR}+1\right)}^{2}-8(R_{NIR}-R_{R})}]/2$	[38]
Difference Vegetation Index (DVI)	$R_{NIR}-R_{R}$	[39]
Green Normalized Difference Vegetation Index (GNDVI)	$\left(R_{NIR}-R_{G})/(R_{NIR}+R_{G}\right)$	[40]
Green Chlorophyll Vegetation Index (GCVI)	$\frac{R_{NIR}}{R_{G}-1}$	[41]
Nonlinear Vegetation Index (NLI)	$\frac{{R_{NIR}}^{2}-R_{RE}}{{R_{NIR}}^{2}+R_{RE}}$	[42]
Ratio Vegetation Index (RVI)	$R_{NIR}/R_{R}$	[43]
Renormalized Difference Vegetation Index (RDVI)	$(R_{NIR}-R_{R})/\sqrt{R_{NIR}+R_{R}}$	[44]
Modified Ratio Vegetation Index (MSR)	$(R_{NIR}/R_{R}-1)/\sqrt{R_{NIR}/R_{R}+1}$	[45]
Normalized Difference Red Edge Index (NDRE)	$\frac{R_{NIR}-R_{RE}}{R_{NIR}+R_{RE}}$	[40]
Triangular Vegetation Index (TVI)	$60(R_{NIR}-R_{G})-100(R_{R}-R_{G})$	[46]
Normalized Difference Vegetation Index (NDVI)	$\left(R_{NIR}-R_{R})/(R_{NIR}+R_{R}\right)$	[47]
Enhanced Vegetation Index (EVI)	$2.5(R_{NIR}-R_{R})/(R_{NIR}+6R_{R}-7.5R_{B}+1)$	[36]

Notes: In the table, $R_{B}$, $R_{G}$, $R_{R}$, $R_{RE}$, $R_{NIR}$ represent the band reflectance of blue, green, red, red edge, and near-infrared bands, respectively, X represents the optimized value to reduce soil background effects, set as 0.16 in this study. The near-infrared bands used in this study were 800 and 900 nm, chosen based on their high correlation with the samples.

**Table 2:** Texture-feature calculation formula.

Textural Features	Formula
Mean, *Mea*	$Mea=\sum_{i,j=1}^{G}\left(iP(i,j)\right)$
Variance, *Var*	$Var=\sum_{i=1}^{G}\sum_{j=1}^{G}{\left(i-u\right)}^{2}P(i,j)$
Homogeneity, *Hom*	$Hom=\sum_{i=1}^{G}\sum_{j=1}^{G}\frac{P(i,j)}{1+{\left(i-j\right)}^{2}}$
Contrast, *Con*	$Con=\sum_{i=1}^{G}\sum_{j=1}^{G}{\left(i-j\right)}^{2}P(i,j)$
Dissimilarity, *Dis*	$Dis=\sum_{i=1}^{G}\sum_{j=1}^{G}P(i,j)\left|i-j\right|$
Entropy, *Ent*	$Ent=\sum_{i=1}^{G}\sum_{j=1}^{G}P(i,j)\log P(i,j)$
Second moment, *Sec*	$\mathrm{Sec}=\sum_{i=1}^{G}\sum_{j=1}^{G}P^{2}(i,j)$
Correlation, *Cor*	$Cor=\sum_{i=1}^{G}\sum_{j=1}^{G}\frac{\left(i-MEA_{j})(j-MEA_{i})P(i,j\right)}{\sqrt{VAR_{i}}\sqrt{VAR_{j}}}$

**Table 3:** Correlation coefficient between vegetation index and SMC in different soil layers under soybean cultivation (** significant at *p* < 0.01).

Soil Depth	Vegetation Index	Correlation Coefficient
0–20 cm	SAVI	0.303 **
	MSAVI	0.318 **
	DVI	0.412 **
	RDVI	0.309 **
	MSR	−0.661 **
	TVI	0.351 **
	EVI	0.312 **
	NLI	0.501 **
	GCVI	−0.667 **
20–40 cm	MSAVI	0.300 **
	DVI	0.397 **
	MSR	−0.657 **
	TVI	0.336 **
	NLI	0.484 **
	GCVI	−0.659 **
40–60 cm	DVI	0.288 **
	MSR	−0.510 **
	NLI	0.328 **
	GCVI	−0.517 **

**Table 4:** Correlation coefficient between texture features and SMC in different soil layers under soybean cultivation (* significant at *p* < 0.05, ** significant at *p* < 0.01).

Soil Depth	Texture Features	Correlation Coefficients					
		Band1	Band2	Band3	Band4	Band5	Band6
0–20 cm	Mean	0.510 **	0.485 **	0.555 **	0.487 **	0.384 **	0.393 **
	Variance	0.470 **	0.435 **	0.575 **	0.521 **	0.407 **	0.427 **
	Homogeneity	0.493 **	0.546 **	0.530 **	0.548 **	0.569 **	0.557 **
	Contrast	0.456 **	0.418 **	0.557 **	0.494 **	0.360 **	0.379 **
	Dissimilarity	0.522 **	0.475 **	0.530 **	0.504 **	0.458 **	0.478 **
	Entropy	0.506 **	0.495 **	0.505 **	0.493 **	0.482 **	0.486 **
	Second Moment	0.566 **	0.579 **	0.568 **	0.579 **	0.585 **	0.580 **
	Correlation	0.553 **	0.548 **	0.566 **	0.548 **	0.557 **	0.542 **
20–40 cm	Mean	0.597 *	0.644 *	0.618 *	0.606 *	0.249	0.259
	Variance	0.578 *	0.559 *	0.398 *	0.513 *	0.485 *	0.505 *
	Homogeneity	0.554 *	0.328 *	0.268 *	0.164	0.429 *	0.424 *
	Contrast	0.585 *	0.581 *	0.439 *	0.531 *	0.492 *	0.506 *
	Dissimilarity	0.581 *	0.223	0.046	0.170	0.505 *	0.523 *
	Entropy	0.389 *	0.210	0.229	0.138	0.251	0.248
	Second Moment	0.457 *	0.285 *	0.399 *	0.358 *	0.398 *	0.396 *
	Correlation	0.540 **	0.534 **	0.554 **	0.535 **	0.543 **	0.530 **
40–60 cm	Mean	0.345 **	0.403 **	0.519 **	0.348 **	0.198	0.212 *
	Variance	0.250 *	0.235 *	0.346 **	0.286 **	0.180	0.195
	Homogeneity	0.380 **	0.412 **	0.381 **	0.431 **	0.454 **	0.443 **
	Contrast	0.241 *	0.228 *	0.339 **	0.268 **	0.141	0.155
	Dissimilarity	0.300 **	0.276 **	0.326 **	0.286 **	0.244 *	0.259 **
	Entropy	0.333 **	0.329 **	0.341 **	0.324 **	0.311 **	0.314 **
	Second Moment	0.427 **	0.432 **	0.416 **	0.437 **	0.447 **	0.444 **
	Correlation	0.383 **	0.376 **	0.402 **	0.380 **	0.384 **	0.375 **

**Table 5:** The texture index extracted by random combination and the correlation coefficient with soybean SMC (** significant at *p* < 0.01).

Soil Depth	Texture Features Extracted by Random Combination	Maximum Correlation Coefficient	
		Correlation Coefficient	Texture Feature Combination
0–20 cm	NDTI	0.471 **	(Hom6, Sec5)
	DTI	0.585 **	(Dis2, Var3)
	RTI	0.504 **	(Mea4, Cor3)
	NTI	−0.626 **	(Var4, Con5)
	RDTI	0.646 **	(Cor3, Ent5)
	RATI	0.624 **	(Sec5, Cor3)
20–40 cm	NDTI	0.488 **	(Hom6, Sec5)
	DTI	0.502 **	(Dis5, Var3)
	RTI	0.492 **	(Hom6, Sec5)
	NTI	−0.583 **	(Sec5, Hom5)
	RDTI	0.540 **	(Cor3, Ent5)
	RATI	0.538 **	(Sec5, Cor3)
40–60 cm	NDTI	0.440 **	(Con5, Var6)
	DTI	0.525 **	(Ent5, Mea3)
	RTI	0.451 **	(Con5, Var6)
	NTI	−0.550 **	(Mea3, Mea2)
	RDTI	0.530 **	(Var4, Con5)
	RATI	0.534 **	(Mea3, Mea3)

**Table 6:** Validation statistics of soybean SMC models by using single-input variable type.

Variable Types	Metrics	0–20 cm			20–40 cm			40–60 cm		
		RF	XGBoost	GA-BP	RF	XGBoost	GA-BP	RF	XGBoost	GA-BP
VIs	R^2^	0.548	0.683	0.592	0.426	0.548	0.468	0.401	0.456	0.426
	RMSE (%)	0.711	0.631	0.640	0.936	0.631	0.760	0.927	0.789	0.795
	MRE (%)	3.444	2.803	3.017	3.443	2.705	3.248	3.644	2.910	2.925
TF	R^2^	0.356	0.421	0.389	0.305	0.374	0.339	0.287	0.354	0.319
	RMSE (%)	0.981	0.854	0.911	1.021	0.924	1.011	1.123	1.011	1.098
	MRE (%)	7.011	6.758	6.981	7.214	6.985	7.054	7.308	7.011	7.159
TIs	R^2^	0.464	0.561	0.521	0.364	0.415	0.391	0.343	0.389	0.368
	RMSE (%)	0.768	0.714	0.744	0.788	0.735	0.764	0.801	0.749	0.788
	MRE (%)	6.214	6.051	6.112	6.521	6.314	6.441	6.601	6.412	6.501
TVIs	R^2^	0.504	0.601	0.554	0.415	0.452	0.436	0.384	0.431	0.401
	RMSE (%)	0.666	0.644	0.681	0.700	0.671	0.684	0.722	0.695	0.711
	MRE (%)	5.748	5.549	5.694	6.012	5.814	5.911	6.124	5.947	6.102

**Table 7:** Validation statistics of soybean SMC models by using multiple-input variable type.

Variable Types	Metrics	0–20 cm			20–40 cm			40–60 cm		
		RF	XGBoost	GA-BP	RF	XGBoost	GA-BP	RF	XGBoost	GA-BP
VIs + TIs	R^2^	0.664	0.751	0.713	0.631	0.695	0.653	0.492	0.567	0.519
	RMSE (%)	0.597	0.476	0.591	0.809	0.650	0.801	1.001	0.863	1.056
	MRE (%)	2.752	2.199	2.873	3.145	2.587	3.209	4.046	3.598	3.939
TVIs + VIs	R^2^	0.582	0.676	0.641	0.559	0.606	0.571	0.422	0.479	0.434
	RMSE (%)	0.631	0.519	0.638	0.855	0.676	0.827	1.053	0.886	1.071
	MRE (%)	2.939	2.269	2.966	3.289	2.736	3.389	4.253	3.751	4.063
TVIs + TIs	R^2^	0.570	0.654	0.634	0.529	0.599	0.568	0.400	0.474	0.432
	RMSE (%)	0.657	0.531	0.674	0.872	0.720	0.894	1.046	0.925	1.128
	MRE (%)	3.012	2.457	3.089	3.391	2.821	3.464	4.313	3.808	4.225
VIs + TIs + TVIs	R^2^	0.689	0.780	0.740	0.654	0.715	0.676	0.520	0.589	0.545
	RMSE (%)	0.548	0.437	0.543	0.743	0.592	0.734	0.937	0.793	0.981
	MRE (%)	2.241	1.667	2.344	2.609	2.112	2.717	3.563	3.080	3.430
VIs + TIs + TF	R^2^	0.605	0.694	0.658	0.574	0.627	0.593	0.437	0.502	0.463
	RMSE (%)	0.582	0.473	0.582	0.781	0.622	0.764	0.969	0.825	1.016
	MRE (%)	2.823	2.240	2.903	3.169	2.712	3.298	4.121	3.665	4.030
VIs + Tis + TF + TVIs	R^2^	0.535	0.609	0.594	0.506	0.560	0.530	0.363	0.442	0.389
	RMSE (%)	0.696	0.561	0.709	0.930	0.750	0.921	1.086	0.982	1.151
	MRE (%)	3.278	2.684	3.377	3.574	3.168	3.710	4.593	4.096	4.511

## Data Availability

Data are contained within the article.

## References

[1] Tang Z., Zhang W., Huang X., Zhang F., Chen J. (2024). Estimation Model of Soybean Yield Based on Ground Hyperspectral Remote Sensing. Trans. Chin. Soc. Agric. Mach..

[2] Wang R., Lin F., Feng K. (2024). Soybean overweight shock (SOS): The impact of trade liberalization in China on overweight prevalence. China Econo. Rev..

[3] Xu Z., Ren T., Marowa P., You X., Lu X., Li Y., Zhang C. (2020). Establishment of a Cultivation Mode of Glycine soja, the Bridge of Phytoremediation and Industrial Utilization. Agronomy.

[4] Huang L., Cai Y., Fang F., Huang T., Zhao M., Zhao Q., Van der Meeren P. (2024). Recent advance in the valorization of soy-based by-products: Extraction, modification, interaction and applications in the food industry. Food Hydrocoll..

[5] Hou D., Bolan N.S., Tsang D.C., Kirkham M.B., O’Connor D. (2020). Sustainable soil use and management: An interdisciplinary and systematic approach. Sci. Total Environ..

[6] Guo M. (2021). Soil Health Assessment and Management: Recent Development in Science and Practices. Soil Syst..

[7] Liu L., Gudmundsson L., Hauser M., Qin D., Li S., Seneviratne S.I. (2020). Soil moisture dominates dryness stress on ecosystem production globally. Nat. Commun..

[8] Chakraborty D., Nagarajan S., Aggarwal P., Gupta V.K., Tomar R.K., Garg R.N., Sahoo R.N., Sarkar A., Chopra U.K., Sundara Sarma K.S. (2008). Effect of mulching on soil and plant water status, and the growth and yield of wheat (*Triticum aestivum* L.) in a semi-arid environment. Agric. Water Manag..

[9] Fu Z., Ciais P., Feldman A.F., Gentine P., Makowski D., Prentice I.C., Stoy P., Bastos A., Wigneron J.P. (2022). Critical soil moisture thresholds of plant water stress in terrestrial ecosystems. Sci. Adv..

[10] Fahad M., Ahmad I., Rehman M.M., Waqas M.M. (2019). Regional wheat yield estimation by integration of remotely sensed soil moisture into a crop model. Can. J. Remote Sens..

[11] Zhang B., Huang J., Dai T., Jing S., Hua Y., Zhang Q., Liu H., Wu Y., Zhang Z., Chen J. (2024). Assessing accuracy of crop water stress inversion of soil water content all day long. Precis. Agric..

[12] Zhou H., Geng G., Yang J., Hu H., Sheng L., Lou W. (2022). Improving Soil Moisture Estimation via Assimilation of Remote Sensing Product into the DSSAT Crop Model and Its Effect on Agricultural Drought Monitoring. Remote Sens..

[13] Yang M., Wang G., Lazin R., Shen X., Anagnostou E. (2021). Impact of planting time soil moisture on cereal crop yield in the Upper Blue Nile Basin: A novel insight towards agricultural water management. Agric. Water Manag..

[14] Du R., Wu J., Tian F., Yang J., Hua X., Chen M., Zhao B., Lin J. (2023). Reversal of soil moisture constraint on vegetation growth in North China. Sci. Total Environ..

[15] Tang Z., Wang X., Xiang Y., Liang J., Guo J., Li W., Li Z., Zhang F. (2024). Application of hyperspectral technology for leaf function monitoring and nitrogen nutrient diagnosis in soybean (*Glycine max* L.) production systems on the Loess Plateau of China. Eur. J. Agron..

[16] Deng K.A.K., Lamine S., Pavlides A., Petropoulos G.P., Srivastava P.K., Bao Y., Hristopulos D., Anagnostopoulos V. (2019). Operational Soil Moisture from ASCAT in Support of Water Resources Management. Remote Sens..

[17] Tang Z., Lu J., Xiang Y., Shi H., Sun T., Zhang W., Wang H., Zhang X., Li Z., Zhang F. (2024). Farmland mulching and optimized irrigation increase water productivity and seed yield by regulating functional parameters of soybean (*Glycine max* L.) leaves. Agric. Water Manag..

[18] Edokossi K., Calabia A., Jin S., Molina I. (2020). GNSS-Reflectometry and Remote Sensing of Soil Moisture: A Review of Measurement Techniques, Methods, and Applications. Remote Sens..

[19] Delavarpour N., Koparan C., Nowatzki J., Bajwa S., Sun X. (2021). A Technical Study on UAV Characteristics for Precision Agriculture Applications and Associated Practical Challenges. Remote Sens..

[20] Liu Z., Li J. (2023). Application of Unmanned Aerial Vehicles in Precision Agriculture. Agriculture.

[21] Peng X., Chen D., Zhou Z., Zhang Z., Xu C., Zha Q., Wang F., Hu X. (2022). Prediction of the Nitrogen, Phosphorus and Potassium Contents in Grape Leaves at Different Growth Stages Based on UAV Multispectral Remote Sensing. Remote Sens..

[22] Miller G.J., Morris J.T., Wang C. (2019). Estimating Aboveground Biomass and Its Spatial Distribution in Coastal Wetlands Utilizing Planet Multispectral Imagery. Remote Sens..

[23] Zhang L., Wang A., Zhang H., Zhu Q., Zhang H., Sun W., Niu Y. (2024). Estimating Leaf Chlorophyll Content of Winter Wheat from UAV Multispectral Images Using Machine Learning Algorithms under Different Species, Growth Stages, and Nitrogen Stress Conditions. Agriculture.

[24] Zhu S., Cui N., Guo L., Jin H., Jin X., Jiang S., Wu Z., Lv M., Chen F., Liu Q. (2024). Enhancing precision of root-zone soil moisture content prediction in a kiwifruit orchard using UAV multi-spectral image features and ensemble learning. Comput. Electron. Agric..

[25] Zhang Y., Yang W., Sun Y., Chang C., Yu J., Zhang W. (2021). Fusion of Multispectral Aerial Imagery and Vegetation Indices for Machine Learning-Based Ground Classification. Remote Sens..

[26] Orsini R., Fiorentini M., Zenobi S. (2020). Evaluation of Soil Management Effect on Crop Productivity and Vegetation Indices Accuracy in Mediterranean Cereal-Based Cropping Systems. Sensors.

[27] Naeem S., Ali A., Chesneau C., Tahir M.H., Jamal F., Sherwani R.A.K., Ul Hassan M. (2021). The Classification of Medicinal Plant Leaves Based on Multispectral and Texture Feature Using Machine Learning Approach. Agronomy.

[28] Yang W., Li Z., Chen G., Cui S., Wu Y., Liu X., Meng W., Liu Y., He J., Liu D. (2024). Soybean (*Glycine max* L.) Leaf Moisture Estimation Based on Multisource Unmanned Aerial Vehicle Image Feature Fusion. Plants.

[29] Leng P., Song X., Duan S., Li Z. (2016). A practical algorithm for estimating surface soil moisture using combined optical and thermal infrared data. Int. J. Appl. Earth Obs..

[30] Wigmore O., Mark B., McKenzie J., Michel B., Lautz L. (2019). Sub-metre mapping of surface soil moisture in proglacial valleys of the tropical Andes using a multispectral unmanned aerial vehicle. Remote Sens. Environ..

[31] Marques P., Pádua L., Sousa J.J., Fernandes-Silva A. (2023). Assessing the Water Status and Leaf Pigment Content of Olive Trees: Evaluating the Potential and Feasibility of Unmanned Aerial Vehicle Multispectral and Thermal Data for Estimation Purposes. Remote Sens..

[32] Peng J., Nieto H., Andersen M.N., Korup K., Larsen R., Morel J., Zhou Z., Manevski K. (2023). Accurate estimates of land surface energy fluxes and irrigation requirements from UAV-based thermal and multispectral sensors. ISPRS J. Photogramm..

[33] Ma J., Liu B., Ji L., Zhu Z., Wu Y., Jiao W. (2023). Field-scale yield prediction of winter wheat under different irrigation regimes based on dynamic fusion of multimodal UAV imagery. Int. J. Appl. Earth Obs..

[34] Peng X., Ma Y., Sun J., Chen D., Zhen J., Zhang Z., Hu X., Wang Y. (2024). Grape leaf moisture prediction from UAVs using multimodal data fusion and machine learning. Precis. Agric..

[35] Cogato A., Meggio F., Collins C., Marinello F. (2020). Medium-Resolution Multispectral Data from Sentinel-2 to Assess the Damage and the Recovery Time of Late Frost on Vineyards. Remote Sens..

[36] Huete A., Didan K., Miura T., Rodriguez E.P., Gao X., Ferreira L.G. (2002). Overview of the radiometric and biophysical performance of the MODIS vegetation indices. Remote Sens. Environ..

[37] Rondeaux G., Steven M., Baret F. (1996). Optimization of Soil-Adjusted Vegetation Indices. Remote Sens. Environ..

[38] Lee M., Kim S.J., Ha M. (2019). Community greenness and neurobehavioral health in children and adolescents. Sci. Total Environ..

[39] Richardson A.J., Wiegand C.L. (1997). Distinguishing vegetation from soil background information. Photogramm. Eng. Remote Sens..

[40] Gitelson A.A., Merzlyak M.N. (1997). Remote estimation of chlorophyll content in higher plant leaves. Int. J. Remote Sens..

[41] Shuai G., Fowler A., Basso B. (2024). Within-season vegetation indices and yield stability as a predictor of spatial patterns of Maize (*Zea mays* L) yields. Precis. Agric..

[42] Kalouli A.L., Hu H., Webb A.F., Moss L.S., Paiva V. (2023). Curing the SICK and Other NLI Maladies. Comput. Linguist..

[43] Gu Z., Zeng Z., Shi X., Li L., Yu D., Zheng W. (2009). Assessing factors influencing vegetation coverage calculation with remote sensing imagery. Int. J. Remote Sens..

[44] Bagheri N. (2020). Application of aerial remote sensing technology for detection of fire blight infected pear trees. Comput. Electron. Agric..

[45] Xue H.Y., Xu X.G., Zhu Q.Z., Meng Y., Long H.L., Li H.L., Song X.Y., Yang G.J., Yang M., Li Y.F. (2024). Rice yield and quality estimation coupling hierarchical linear model with remote sensing. Comput. Electron. Agric..

[46] Wiegand C.L., Richardson A.J., Kanemasu E.T. (1979). Leaf area index estimates for wheat from LANDSAT and their implications for evapotranspiration and crop modeling. Agron. J..

[47] Rouse J.W., Haas R.H., Schell J.A., Deering D.W., Harlan J.C. (1974). Monitoring the Vernal Advancement of Retrogradation (Green Wave Effect) of Natural Vegetation. https://ntrs.nasa.gov/citations/19750020419.

[48] Jing X., Zou Q., Yan J., Dong Y., Li B. (2022). Remote Sensing Monitoring of Winter Wheat Stripe Rust Based on mRMR-XGBoost Algorithm. Remote Sens..

[49] Pan Y., Ren C., Liang Y., Zhang Z., Shi Y. (2020). Inversion of surface vegetation water content based on GNSS-IR and MODIS data fusion. Satell. Navig..

[50] Liu Z., Ji Y., Ya X., Liu R., Liu Z., Zong X., Yang T. (2024). Ensemble Learning for Pea Yield Estimation Using Unmanned Aerial Vehicles, Red Green Blue, and Multispectral Imagery. Drones.

[51] Gerhards M., Schlerf M., Mallick K., Udelhoven T. (2019). Challenges and Future Perspectives of Multi-/Hyperspectral Thermal Infrared Remote Sensing for Crop Water-Stress Detection: A Review. Remote Sens..

[52] Liang S., Ma W., Sui X., Wang M., Li H. (2023). An Assessment of Relations between Vegetation Green FPAR and Vegetation Indices through a Radiative Transfer Model. Plants.

[53] Tang Z., Guo J., Xiang Y., Lu X., Wang Q., Wang H., Cheng M., Wang H., Wang X., An J. (2022). Estimation of Leaf Area Index and Above-Ground Biomass of Winter Wheat Based on Optimal Spectral Index. Agronomy.

[54] Ma S., Liu S., Gao Z., Wang X., Ma S., Wang S. (2024). Water Deficit Diagnosis of Winter Wheat Based on Thermal Infrared Imaging. Plants.

[55] Lu J., Eitel J., Engels M., Zhu J., Ma Y., Liao F., Zheng H., Wang X., Yao X., Cheng T. (2021). Improving Unmanned Aerial Vehicle (UAV) remote sensing of rice plant potassium accumulation by fusing spectral and textural information. Int. J. Appl. Earth Obs..

[56] Wu G., Fang Y., Jiang Q., Cui M., Li N., Ou Y., Diao Z., Zhang B. (2023). Early identification of strawberry leaves disease utilizing hyperspectral imaging combing with spectral features, multiple vegetation indices and textural features. Comput. Electron. Agric..

[57] Zhou C., Gong Y., Fang S., Yang K., Peng Y., Wu X., Zhu R. (2022). Combining spectral and wavelet texture features for unmanned aerial vehicles remote estimation of rice leaf area index. Front. Plant Sci..

[58] Wang R., Tuerxun N., Zheng J. (2024). Improved estimation of SPAD values in walnut leaves by combining spectral, texture, and structural information from UAV-based multispectral image. Sci. Hortic..

